# Why Organised Cancer Screening Works: Lessons from Slovenia and the Next Phase of Evidence-Based Implementation

**DOI:** 10.2478/sjph-2026-0016

**Published:** 2026-09-01

**Authors:** Mateja Krajc, Urška Ivanuš

**Affiliations:** Institute of Oncology Ljubljana, Zaloška cesta 2, 1000 Ljubljana, Slovenia

**Keywords:** Cancer screening, Organised programmes, Public health, Quality assurance, Slovenia, presejanje za raka, organizirani programi, javno zdravje, zagotavljanje kakovosti, Slovenija

## Abstract

Slovenia's experience shows that cancer screening becomes effective only when a test is embedded in an organised, population-based programme that reaches the target population equitably, assures quality across the whole pathway and uses individual- and programme-level data for improvement. Since the national roll-out of Programme ZORA for cervical cancer screening in 2003, followed by DORA for breast cancer screening in 2008 and Programme Svit for colorectal cancer screening in 2009, Slovenia has built a coherent screening infrastructure with visible population effects. Cervical and colorectal cancer incidence and mortality are decreasing, breast cancers detected through DORA are increasingly diagnosed at a localised stage, and Slovenia ranks among the strongest European performers in screening coverage and policy. This editorial argues that these achievements should be understood not as three isolated programme successes, but as the result of governance, registries, quality assurance, legal foundations, public trust and international alignment. The same principles must now guide the modernisation of existing programmes and the cautious, evidence-based introduction of lung, prostate and gastric cancer screening.

## Izvleček

Slovenska izkušnja kaže, da presejanje za raka postane učinkovito šele takrat, ko je presejalni test umeščen v organiziran populacijski program, ki ciljno populacijo dosega pravično, zagotavlja kakovost na celotni presejalni poti ter uporablja podatke na ravni posameznika in programa za stalno izboljševanje. Od uvedbe državnega programa ZORA za presejanje raka materničnega vratu leta 2003, ki sta mu sledila program DORA za presejanje raka dojk leta 2008 in Program Svit za presejanje raka debelega črevesa in danke leta 2009, je Slovenija vzpostavila povezano presejalno infrastrukturo z vidnimi učinki na populacijski ravni. Incidenca in umrljivost zaradi raka materničnega vratu ter raka debelega črevesa in danke se zmanjšujeta, raki dojk, odkriti v programu DORA, so vse pogosteje diagnosticirani v omejenem stadiju, Slovenija pa se po pregledanosti in presejalnih politikah uvršča med najuspešnejše evropske države. V uvodniku zagovarjamo stališče, da teh dosežkov ne smemo razumeti kot treh ločenih programskih uspehov, temveč kot rezultat upravljanja, presejalnih registrov, zagotavljanja kakovosti, zakonskih podlag, zaupanja javnosti in mednarodne usklajenosti. Ista načela morajo zdaj usmerjati tudi posodobitev obstoječih programov ter premišljeno, na dokazih temelječe uvajanje presejanja za pljučnega raka, raka prostate in raka želodca.

## 1 INTRODUCTION

Organised screening programmes are designed for apparently healthy individuals who do not have symptoms of the disease being screened for. This creates a high ethical and organisational threshold: expected benefits must clearly outweigh potential harms, and these benefits must be achievable in routine health care. A screening test alone cannot guarantee this balance. False-positive results, false reassurance after false-negative results, overdiagnosis, overtreatment, psychological burden and opportunity costs are inherent risks of screening. They are magnified when screening is opportunistic, fragmented and poorly monitored, and reduced when screening is organised, population-based and quality-assured (1,2,3,4).

The classic Wilson-Jungner principles and their contemporary updates therefore, remain highly relevant (1,2,3,4). Modern cancer-screening policy adds explicit governance, call-recall systems, quality assurance, screening registries linked with population and cancer-registry data, monitoring of performance indicators and transparent communication of both benefits and harms (1,2,3,4). Slovenia is a useful case study because it moved from opportunistic screening to organized cervical cancer screening using the public health approach and applied it also to implement organized breast and colorectal cancer screening.

## 2 ORGANISED SCREENING AS PUBLIC-HEALTH INFRASTRUCTURE

The distinction between organised and opportunistic screening algorithm is a fundamental determinant of effectiveness and fairness. Organised screening identifies the target population, invites eligible individuals, defines the screening policy, ensures access to diagnostic assessment and treatment, monitors each step of the pathway, and evaluates outcomes at the population level. Opportunistic screening depends on individual initiative, local practice and variable provider behaviour. It may reach people who are already more health-literate while missing disadvantaged groups and non-responders, and it may lead to both too frequent testing in some people and no testing in others.

In Slovenia, organisation rests on several mutually reinforcing elements: central coordination by designated national institutions; screening registries that support invitation, recall, quality assurance and evaluation; programme guidelines and provider standards; integration of screening, diagnosis, treatment and follow-up; universal access without direct cost for eligible populations; legal and financing foundations; professional education; audit; and communication with the public.

Screening is a continuum of care. A positive screening test without timely, high-quality diagnostic and therapeutic services may produce anxiety and system burden without reducing morbidity or mortality. Conversely, a monitored pathway can turn early detection into a measurable public health benefit (5,6,7,8).

## 3 SLOVENIA'S THREE CANCER SCREENING PROGRAMMES AND THEIR MEASURABLE EFFECTS

Programme ZORA, the national cervical cancer screening programme, has operated nationally since 2003 and currently invites and monitors women aged 20–64 years. Its development was driven by cancer-registry evidence that opportunistic cytology screening had not sufficiently reduced the burden of cervical cancer. Since ZORA became organised, cervical cancer incidence has more than halved, and Slovenia is approaching the World Health Organization (WHO) elimination threshold for cervical cancer as a public-health problem (5, 6).

DORA, the national breast cancer screening programme, started in 2008 and achieved national roll-out in 2017. It invites women aged 50–69 years to quality-assured mammography every 2 years through a network of mobile units and screening and diagnostic centres. In Slovenia, the proportion of breast cancers detected at a localised stage has increased substantially, and approximately 80% of cancers detected in DORA are diagnosed at a localised stage (7, 8, 9, 10). Programmes DORA and ZORA are coordinated by the Institute of Oncology Ljubljana (IOL), Slovenia.

Programme Svit started in 2009 and is coordinated by the National Institute of Public Health, invites men and women aged 50–74 years to biennial colorectal cancer screening using a faecal immunochemical test and after several screening rounds, colorectal cancer has fallen from the most common cancer in Slovenia to a lower rank among all cancers (7, 11, 12).

Cancer-specific mortality has decreased by 5.3% per year in ZORA and by 4.8% per year in the Svit Programme. In the DORA programme, the proportion of cancers detected at a localised stage has increased by one-third nationally over the past ten years, while mortality has decreased by 1.0% per year. Since the DORA programme does not detect precancerous lesions and does not reduce incidence, the full impact of screening on mortality is expected to become evident in just over a decade. International comparisons support this national picture. The EU Country Cancer Profiles Synthesis Report 2025 reported above-average screening coverage in Slovenia: ZORA 74%, Programme Svit 65% and DORA 78%, placing Slovenia second, third and fourth, respectively, among European countries for the corresponding screening programmes (7). For instance, the European Cancer Screening Policy Index 2024 ranked Slovenia first among European countries, with a score of 91.2%, reflecting not only coverage but also governance, registries and quality assurance (13). These rankings should not be read as a reason for complacency. They show what becomes possible when screening is treated as a long-term public-health infrastructure.

## 4 A NEW EUROPEAN PHASE: THE 2022 COUNCIL RECOMMENDATION AND SLOVENIA'S RESPONSE

The 2022 Council Recommendation on cancer screening marked a new European phase (14). It confirmed the importance of organised breast, cervical and colorectal cancer screening, supported human papillomavirus (HPV)-based cervical cancer screening, and invited Member States to explore lung, prostate and, where appropriate, gastric cancer screening in a stepwise, evidence-based manner. Slovenia's response has been both ambitious and cautious (15, 16).

Just before the adoption of the Council Recommendation, in 2020, the Ministry of Health (MoH) established the National Screening Committee to guide the assessment and implementation of new screening initiatives and has laid the groundwork for the implementation of the newly recommended screening programmes (14, 17). The Committee's mandate extends beyond cancer screening and also covers the assessment of screening initiatives for other diseases.

**Figure 1:**
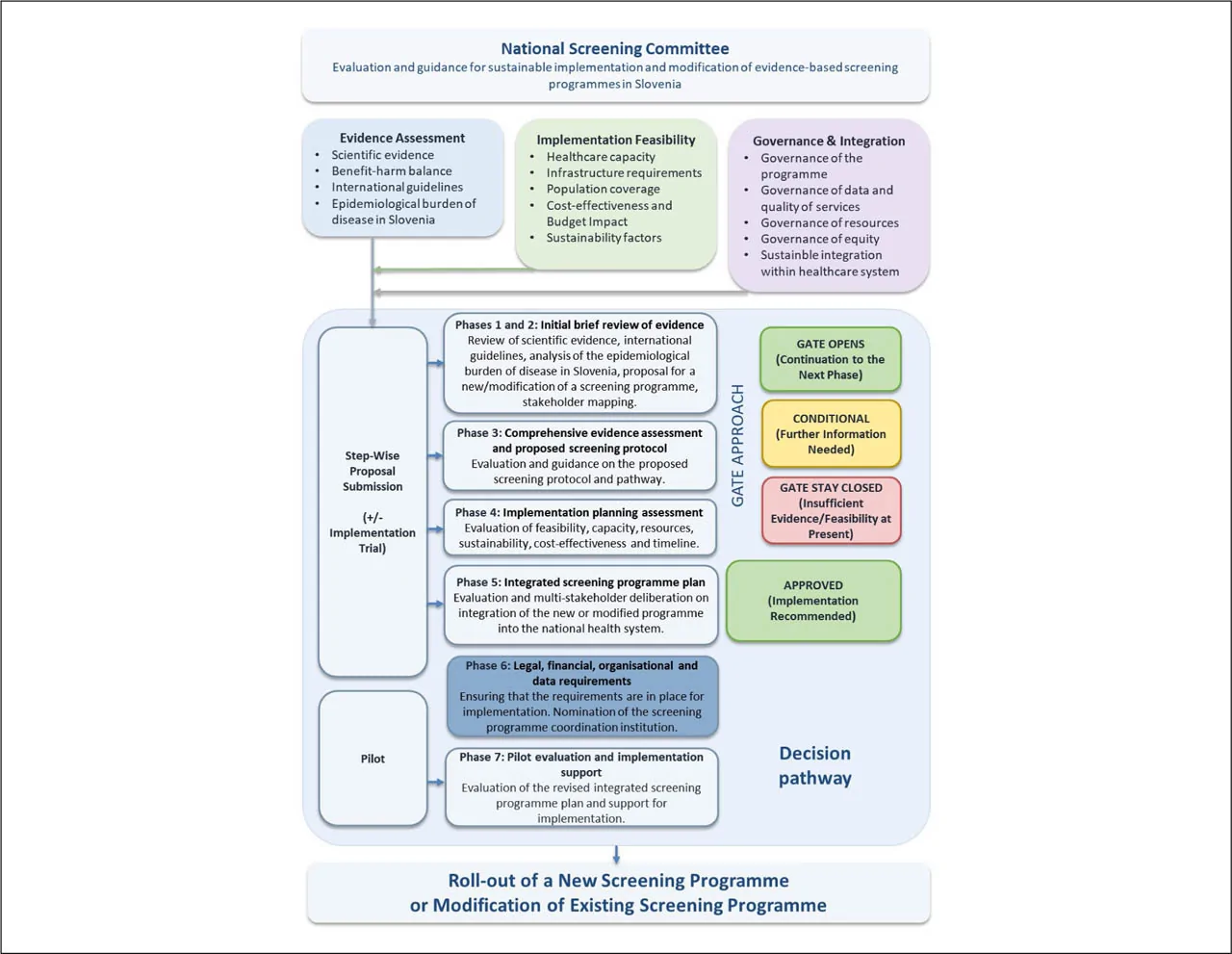
Decision Pathway of the Slovenian National Screening Committee for new screening programmes and modifications of existing programmes. A pragmatic, stepwise framework comprising seven distinct phases, each separated by decision gates. Proposals for new screening programmes are evaluated progressively at each phase to ensure quality, feasibility, sustainability and population-level impact.

The Committee, appointed by the Minister of Health, brings together screening programme leaders, representatives of the MoH, the Health Insurance Institute of Slovenia, the National Cancer Control Programme and non-governmental organisations. Its mandate is to define criteria for new screening programmes, propose experts to assess new initiatives, decide whether proposed programmes are scientifically justified and sufficiently developed for inclusion among compulsory health insurance rights, guide the development of new programmes, and assess proposed changes or improvements to existing ones. In this way, the Committee protects the population from premature, fragmented or inequitable implementation, while enabling timely adoption when evidence, feasibility and system readiness are sufficient. Proposals are expected to move through a structured pathway that considers evidence assessment, international recommendations, stakeholder involvement, clinical guidelines, integration into the health system, quality assurance, registry infrastructure, pilot protocols, resources, cost-effectiveness and legal foundations (Figure 1).

The current Slovenian development projects are aligned with both, the work of the National Screening Committee and the Council Recommendation (14, 17). The project named “ZORA RENEWAL” is preparing for the transition from cytology-based cervical screening to primary HPV screening, with changes in the starting age and screening interval (18). In 2025, a pilot study was launched to assess the expansion of the programme DORA to the 45–49 and 70–74 age groups, while in 2021, a feasibility study of risk-based screening was also conducted (19). The two programmes, named “LUKA” and “PETER” are preparing the professional foundations for lung and prostate cancer screening and have passed the first assessment phase of the National Screening Committee (20, 21). The project named “ProScreen-Slovenia/PRO-PETER”, approved at the end of 2024 and led by the International Agency for Research on Cancer (IARC), in collaboration with Erasmus University Medical Center and the IOL, supports the development of organised prostate cancer screening in Slovenia (22). Its pilot study, PRO-PETER, launched in December 2025, will include 10,000 men aged 50–69 years from the Ljubljana region using a risk-stratified pathway with prostate-specific antigen (PSA) testing, risk assessment, magnetic resonance imaging and biopsy when indicated. By the end of 2026, the new European projects Strengthening the screening of Lung Cancer in Europe (SOLACE+) and PRostate cancer Awareness and Initiative for Screening in the European Union project (PRAISE-U+) will further support Slovenia's first organised lung cancer screening trial and the continuation of prostate cancer screening (23, 24). European and international collaborations, including Improving Cancer Screening in Slovenia (ICSIS) Project, Technical support for the implementation of country-specific recommendations (TSI 2026 flagship), EUROHELICAN project, aimed at accelerating gastric cancer reduction in Europe through Helicobacter pylori eradication, Towards Gastric Cancer Screening Implementation in the European Union (TOGAS) project and Towards improved screening for breast, cervical and colorectal cancer in all of Europe project (EU-TOPIA)/Towards improved screening for breast, cervical and colorectal cancer in Eastern Europe: Equitable, Actionable, Sustainable and Trustworthy project (EU-TOPIA-EAST), support feasibility assessment, pilot testing, modelling, cost-effectiveness analysis and quality improvement. The guiding question is not whether a promising test exists with which we can lower the mortality, but whether the entire pathway can be delivered safely, equitably and sustainably (25,26,27,28).

## 5 LOOKING AHEAD

The future of cancer screening will be defined less by doing more tests than by building smarter, more adaptive and sustainable systems. Cervical cancer screening is becoming a test case for whether Europe can turn screening into elimination. In Slovenia, elimination is realistic if high screening coverage is maintained, HPV vaccination improves, and the transition to high-risk HPV testing is implemented with strong quality assurance.

Screening policy is moving from uniform, age-based programmes towards risk-adapted approaches (29). Risk stratification, including genetics, screening history, lifestyle factors, imaging features, biomarkers and vaccination status, may improve the balance between benefits and harms, but it also introduces a new programme function: risk assessment. This requires robust data infrastructure, transparent communication, and safeguards to ensure that personalisation strengthens rather than fragments population-based screening.

New technologies will further reshape screening pathways. Artificial intelligence may support several points of the screening pathway, including mammography reading, low-dose computed tomography for lung screening, digital cytology, endoscopic quality and workflow management. However, in screening, it must meet the same standards as any intervention offered to healthy people: clinical validation, monitoring in real-world conditions, transparent performance across subgroups, integration into accountable clinical pathways, and evaluation of harms as well as benefits. Ethical considerations, including equity, informed decision-making, data protection, and accountability, must also remain central to its implementation.

Sustainability is already a key implementation challenge. It extends beyond financing to governance, priority-setting, workforce capacity, diagnostic bottlenecks, quality assurance, environmental impact, public trust, leadership and political commitment. Equally important is governance of innovation: transparent, evidence-based decisions on which screening interventions to implement, when to pilot them, when to scale them, and when not yet to proceed. In European Joint Action on Cancer Screening (EUCanScreen), Work Package 4, led by IOL and IARC/WHO, addresses these dimensions to support sustainable, equitable and impactful screening programmes across Europe (30).

Slovenia also continues to develop screening and early detection activities beyond its established cancer screening programmes, by refining cancer screening through risk-stratified approaches, as suggested in 2022 Council Reccomendation (14, 19).

National and research-based initiatives have also been implemented in screening areas beyond cancer, such as the screening and registration of patients with asthma and chronic obstructive pulmonary disease in primary care (31), risk-stratified approaches to breast cancer screening (32), expanded newborn screening supported by advanced molecular diagnostics (33), and participation in European joint actions addressing cardiovascular diseases and diabetes (34). Together, these examples show that Slovenia is actively aligning its screening and prevention strategies with evolving evidence and professional developments in the European Union.

## 6 CONCLUSIONS

Slovenia's cancer screening story is often summarised through three programme names: ZORA, DORA and Svit. Their deeper significance is institutional. Slovenia has built screening as organised public health infrastructure: population-based, data-supported, quality-assured, legally grounded, professionally led, and internationally connected. This infrastructure explains high coverage, measurable reductions in cervical and colorectal cancer burden, favourable stage distribution in screen-detected breast cancer and resilience during periods of disruption.

The future of cancer screening will not be defined simply by doing more tests, but by building better systems. More than ever, mastering screening means mastering system engineering. Artificial intelligence, risk-adapted screening, self-sampling, molecular diagnostics, and multi-cancer early detection tests all offer important opportunities, but their value will depend on robust evidence and careful implementation. Screening programmes must ensure timely diagnostic follow-up, equitable access, sustainable use of resources, transparent communication of benefits and harms, and continuous quality assurance. In this new era, the key measure of success should not be how many people are screened, but how effectively, fairly, and sustainably screening reduces the burden of cancer.

For Slovenia, the next challenge is to remain innovative while preserving the main strength of its screening system: an organised, accountable, high-quality population-based implementation.
